# Stress-Induced Alternative Splice Forms of MDM2 and MDMX Modulate the p53-Pathway in Distinct Ways

**DOI:** 10.1371/journal.pone.0104444

**Published:** 2014-08-08

**Authors:** Aishwarya G. Jacob, Ravi K. Singh, Daniel F. Comiskey, Matthew F. Rouhier, Fuad Mohammad, Thomas W. Bebee, Dawn S. Chandler

**Affiliations:** 1 From the Center for Childhood Cancer at the Research Institute at Nationwide Children's Hospital, Columbus, Ohio, United States of America; 2 The Department of Pediatrics, and Molecular, Cellular and Developmental Biology (MCDB) program, The Ohio State University, Columbus, Ohio, United States of America; 3 Center for RNA Biology, Wexner Medical Center, The Ohio State University, Columbus, Ohio, United States of America; University Hospital of Navarra, Spain

## Abstract

MDM2 and MDMX are the chief negative regulators of the tumor-suppressor protein p53 and are essential for maintaining homeostasis within the cell. In response to genotoxic stress and also in several cancer types, *MDM2* and *MDMX* are alternatively spliced. The splice variants *MDM2-ALT1* and *MDMX-ALT2* lack the p53-binding domain and are incapable of negatively regulating p53. However, they retain the RING domain that facilitates dimerization of the full-length MDM proteins. Concordantly, MDM2-ALT1 has been shown to lead to the stabilization of p53 through its interaction with and inactivation of full-length MDM2. The impact of MDM2-ALT1 expression on the p53 pathway and the nature of its interaction with MDMX remain unclear. Also, the role of the architecturally similar MDMX-ALT2 and its influence of the MDM2-MDMX-p53 axis are yet to be elucidated. We show here that MDM2-ALT1 is capable of binding full-length MDMX as well as full-length MDM2. Additionally, we demonstrate that MDMX-ALT2 is able to dimerize with both full-length MDMX and MDM2 and that the expression of MDM2-ALT1 and MDMX-ALT2 leads to the upregulation of p53 protein, and also of its downstream target p21. Moreover, MDM2-ALT1 expression causes cell cycle arrest in the G1 phase in a p53 and p21 dependent manner, which is consistent with the increased levels of p21. Finally we present evidence that MDM2-ALT1 and MDMX-ALT2 expression can activate subtly distinct subsets of p53-transcriptional targets implying that these splice variants can modulate the p53 tumor suppressor pathway in unique ways. In summary, our study shows that the stress-inducible alternative splice forms MDM2-ALT1 and MDMX-ALT2 are important modifiers of the p53 pathway and present a potential mechanism to tailor the p53-mediated cellular stress response.

## Introduction

The tumor-suppressor protein p53 is a transcription factor crucial for maintaining genomic integrity and for inducing cell-cycle arrest or cell-death pathways in the face of insurmountable cellular insult [1]. Under normal physiological conditions, p53 activity and levels are kept under tight control mainly by the Murine Double Minute (MDM) proteins. MDM2 is an E3 ubiquitin ligase that binds and polyubiquitinates p53 thereby targeting p53 for proteasome-mediated degradation [2]–[7]. Additionally, the binding of MDM2 to p53 blocks the latter's transcriptional activity. MDMX (also known as MDM4), a close family member of MDM2, is also involved in the negative regulation of p53. Although it lacks E3 ligase activity, it is capable of forming either homodimers or heterodimers with MDM2, which inhibit p53's transcriptional activity or aid in the ubiquitination of p53 [8]–[14]. Interestingly, MDM2 regulates its own levels and also that of MDMX via its E3 ubiquitin ligase activity [6], [15]. When over-expressed, MDM2 and MDMX are oncogenic in nature and lead to tumorigenesis by suppressing the activity of p53 and allowing uncontrolled proliferation [9], [16]–[20].

Under conditions necessitating p53 activation, the interaction of MDM2 with p53 is disrupted through several tightly regulated post-translational events targeting these proteins [21]–[23]. Interestingly, in addition to protein modifications, alternative splice forms of MDM2 also play an important role in the activation of p53. At least 10 bona fide splice variants of *MDM2* have been described in different cancer types and in response to stress, whose functions differ from the canonical role of full-length MDM2 in p53-regulation [24]. For instance, splice variants MDM2-ALT2 (MDM2-A, which contains exons 3, 10, 11, and 12) and MDM2-ALT3 (MDM2-C, which contains exons 3, 4, 10, 11, and 12) are incapable of binding and targeting p53 for degradation [25], [26]. In addition, MDM2-ALT1 (MDM2-B, which contains solely exons 3 and 12) and MDM2-ALT2 also sequester full-length MDM2 in the cytoplasm, in effect, stabilizing p53 [24], [26]–[28]. In response to genotoxic stress such as UV irradiation or cisplatinum treatment, the predominant splice variant generated, MDM2-ALT1, also lacks the p53-binding domain but retains the RING domain required for dimerization [24], [29]–[31]. Functionally, MDM2-ALT1 has been shown to interact with and inactivate full-length MDM2 leading to the stabilization of p53 [29], [30], [32], [33]. Curiously, *MDM2-ALT1* is constitutively expressed in several tumor types [28], [34]–[43] and has also been shown to have tumorigenic properties in *in vitro* and *in vivo* systems [43]–[45], a function that directly contrasts its role in upregulation of tumor-suppressor p53. However, a recent study in colorectal tumors demonstrated that constitutive expression of MDM2-ALT1 in tumors with gain-of-function mutant p53 results in the stabilization of the dominant-negative, oncogenic forms of p53 as a result of MDM2 inactivation thereby leading to tumorigenesis [32]. This raises the possibility that in cancer types with mutant p53, MDM2-ALT1 could indeed play the role of an oncogene by altering the activity of its own full-length counterpart. However, it is curious that MDMX, also a potent p53 inhibitor, is unable to inactivate mutant p53 in MDM2-ALT1 expressing cells. Hence, the role of MDMX in the context of cancers presenting with *MDM2-ALT1* remains unclear.

Adding another layer of complexity is the fact that alternative splicing of *MDMX* also occurs in response to genotoxic stress. Particularly, the occurrence of cancer-associated splice variants, *MDMX-S* (that possesses high affinity for p53) and *MDMX-ALT2* (that lacks the p53-binding domain), has been reported upon cisplatinum treatment [29], [43], [46], [47]. While the role of MDMX-S in tumorigenesis as a potent inhibitor of the p53 tumor-suppressor pathway is well understood [48]–[51], the role of MDMX-ALT2 in cancer is not clear. MDMX-ALT2 is architecturally similar to MDM2-ALT1 in that it lacks the ability to bind p53. However, it has been shown to be strongly associated with metastatic pediatric rhabdomyosarcoma (RMS) and has been demonstrated to possess tumorigenic properties *in vitro* [43]. Moreover, *MDMX-ALT2* and *MDM2-ALT1* are coincident in ∼ 24% RMS tumors, however, their role in RMS pathogenesis is not known [43].

The impact that MDM2-ALT1 and MDMX-ALT2 have on the p53 pathway and on their full-length counterparts has remained unclear. By overexpressing MDM2-ALT1, we demonstrate that it interacts with full-length MDMX and MDM2 and also leads to increased levels of the p53. Similarly, MDMX-ALT2 also dimerizes with both full-length MDMX and MDM2, and also leads to increased p53 indicating overlapping roles for MDM2 and MDMX splice variants. Furthermore, our study is the first to illustrate that MDMX-ALT2 and MDM2-ALT1 can modulate the transcriptional activity of the stabilized p53 suggesting a function of these splice variants in adjusting stress response and tumorigenesis.

## Results

### MDM2-ALT1 interacts with full-length MDMX and MDM2

**Figure 1:**
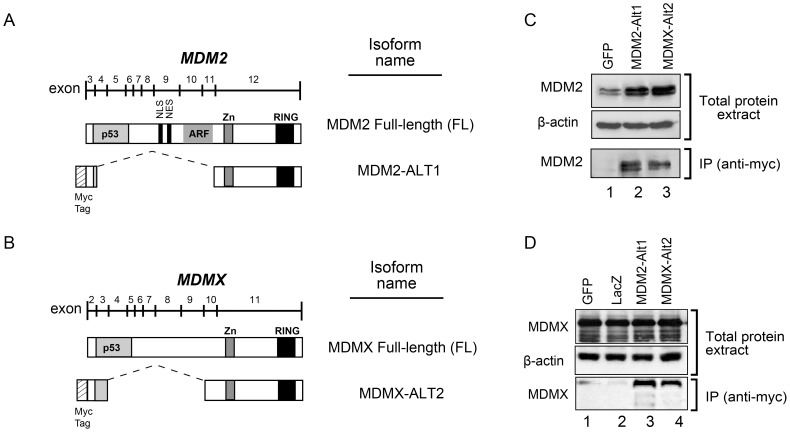
MDM2-ALT1 and MDMX-ALT2 interact with full-length MDM2 and MDMX. A. Full-length MDM2 is encoded by exons 3 to 12 of the *MDM2* gene and consists of the N-terminal p53-binding domain, the nuclear localization (NLS) and export signals (NES), the central ARF binding and Zinc finger domains and the C-terminal RING domain. *MDM2-ALT1* comprises only exons 3 and 12 spliced together and the protein lacks the p53-binding domain. However, it retains the RING domain. **B.** Full-length MDMX, a close family member of MDM2, also comprises an N-terminal p53-binding domain, a central Zinc finger domain and a C-terminal RING domain and is encoded by exons 2 to 11 of the *MDMX* gene. *MDMX-ALT2* consists of exons 2,3,10 and 11 and the protein is architecturally similar to MDM2-ALT1 in that it lacks the p53-binding domain but retains the RING domain. **C.** Myc-tagged constructs of LacZ, MDM2-ALT1 or MDMX-ALT2 were transfected into MCF7 cells. Immunoprecipitation of the myc-tagged proteins revealed the specific binding of full-length MDM2 to MDM2-ALT1 and MDMX-ALT2 and not to negative control protein myc-LacZ (compare lanes 2 and 3 to lane 1). Experiments were repeated a minimum of three times and consistent results were observed. Representative gel images are presented in the figure. **D.** Myc-tagged MDM2-ALT1 and MDMX-ALT2 co-immunoprecipitate with full-length MDMX while the negative control protein myc-LacZ does not interact with MDMX (compare lanes 2 and 3 to lane 1). These results were observed in two independent trials and representative images are shown.

MDM2-ALT1, a major stress- inducible splice variant of MDM2 lacks a p53-binding domain but contains an intact RING domain ([36] and fig 1A). The RING domain facilitates the homodimerization of MDM2 and also hetero-dimerization with MDMX [14], [52]. Previous reports have demonstrated the interaction of MDM2-ALT1 with full-length MDM2, which potentially affects the functions of MDM2 [30], [32]. We wanted to test the possibility that MDM2-ALT1 could also directly form heterodimers with MDMX. To this end, we transfected MCF7 cells with plasmids expressing myc-MDM2-ALT1 or a negative control myc-GFP and performed an immunoprecipitation of the myc-tagged proteins. The immunoprecipitated samples were then probed with antibodies specific for MDM2 or MDMX to identify interaction of MDM2-ALT1 and MDMX-ALT2 with the endogenous isoforms of MDM2 and MDMX (Fig 1 and S1). Consistent with previous reports, we observed the direct interaction of MDM2-ALT1 with full-length MDM2 (Fig 1C and S1A). We additionally performed reciprocal immunoprecipitation of MDM2 to validate interaction of endogenous MDM2 with MDM2-ALT1 in cells expressing control (myc-LacZ) or myc-MDM2-ALT1. Results indicate that myc-MDM2-ALT1 interacted with endogenous MDM2 (Figure S2). We also observed that MDM2-ALT1 interacts directly with full-length MDMX (Fig 1D and S1B). The specificity of these interactions is demonstrated by the fact that myc-tagged LacZ or GFP did not co-immunoprecipitate with either endogenous MDM2 or MDMX (Fig 1C and 1D).

### MDMX-ALT2 dimerizes with MDM2 and MDMX

It has previously been shown that in response to genotoxic stress such as UV and cisplatinum treatment, *MDMX* is also alternatively spliced to create a damage-induced isoform, *MDMX-ALT2* [29], [43], [46], [47]. Similar to MDM2-ALT1, MDMX-ALT2 lacks the p53-binding domain but retains the RING domain (Fig 1B). To test the possibility that MDMX-ALT2 can similarly dimerize with full-length MDMX and MDM2, we over-expressed myc-tagged MDMX-ALT2 in MCF7 cells. The immunoprecipitated myc-MDMX-ALT2 was blotted with specific antibodies against MDM2 or MDMX to assess its interactions with endogenous MDM2 and MDMX isoforms (Fig 1 and S1). Indeed, we observed that myc-tagged MDMX-ALT2 co-immunoprecipitated with full-length MDM2 (Fig 1C and S1A) and MDMX (Fig 1D and S1B). Additionally, we verified interaction of MDM2 with MDMX-ALT2 by reciprocal co-immunoprecipitation of endogenous MDM2 in cultures expressing control (myc-LacZ) or myc-MDMX-ALT2. Results indicated that myc-MDMX-ALT2 interacted with endogenous MDM2 (Figure S2). Importantly, myc-tagged LacZ used as a negative control in these experiments failed to co-immunoprecipitate either MDM2 or MDMX (Fig 1C, D and S1).

### MDM2-ALT1 and MDMX-ALT2 expression stabilizes p53

**Figure 2:**
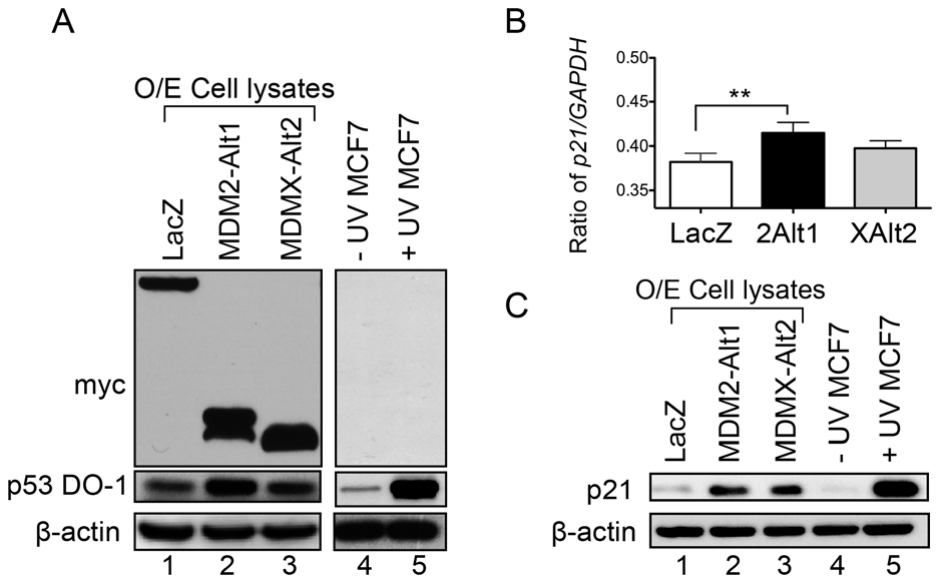
MDM2-ALT1 and MDMX-ALT2 expression causes upregulation of p53 and its downstream target p21. **A.** The over-expression (O/E cell lysates) of the myc-tagged LacZ, MDM2-ALT1 or MDMX-ALT2 was confirmed using anti-myc tag antibody in the MCF7 cells that were transfected with the corresponding expression constructs (top panel). The level of p53 protein was examined in these samples using the anti-p53 antibody and an upregulation of p53 protein was observed upon MDM2-ALT1 or MDMX-ALT2 over-expression compared to LacZ expressing cells although the increase is more modest in MDMX-ALT2 over-expression (lanes 2 and 3 compared to lane 1). The positive control, UVC (50 J/m^2^) irradiated MCF7 cells show a strong upregulation of p53 protein levels in response to the stress when compared to untreated cells (lanes 4 and 5). β-actin was used as loading control. A minimum of three independent experiments was performed and representative gel images are shown. **B.** *p21* expression at the mRNA level was examined using quantitative real-time PCR and *GAPDH* levels were used as the endogenous control. The ratio of *p21* to *GAPDH* is represented graphically and the error bars represent standard deviations from at least 3 independent experiments. MCF7 cells over-expressing MDM2-ALT1 (2Alt1) show statistically significant increase in *p21* transcript levels compared to LacZ expressing cells (p<0.01). The cells expressing MDMX-ALT2 (XAlt2) did not show statistically significant changes in *p21* expression at the mRNA level. **C.** The levels of p21 protein in the MCF7 cells transfected with myc-tagged LacZ, MDM2-ALT1 or MDMX-ALT2 was examined using anti-p21 antibody. Both MDM2-ALT1 and MDMX-ALT2 over-expression lead to upregulation of p21 protein levels compared to LacZ over-expression (compare lanes 2 and 3 with lane 1). A minimum of three independent experiments was performed and consistent results observed. Representative images are shown here. Additionally, UVC-irradiated MCF7 cells were used as positive control and show an upregulation of p21 compared to untreated cells (lanes 4 and 5).

Since MDM2-ALT1 and MDMX-ALT2 interact with full-length MDM2 and MDMX, it is possible that the formation of such complexes can impede the functions of these proteins. Moreover, MDM2-ALT1 expression has been shown previously to lead to the accumulation of p53 due to sequestration of MDM2 in the cytoplasm [29], [30], [32]. We hypothesized that MDMX-ALT2 expression could increase p53 levels in a similar manner. To test this, we examined the expression of p53 in MCF7 cells that were transfected with a negative control (LacZ), MDMX-ALT2 or MDM2-ALT1. As expected, MDM2-ALT1 expression caused a substantial increase in the levels of p53 protein compared to LacZ over-expression (Fig 2A p53 panel compare lanes 1 and 2). MDMX-ALT2 expression also resulted in an increase in the p53 protein levels compared to LacZ over-expression (Fig 2A p53 panel compare lanes 1 and 3). However, this effect was more moderate compared to the MDM2-ALT1-mediated upregulation of p53 protein (Fig 2A p53 panel). As a positive control for the accumulation of p53 protein, we compared p53 levels in whole cell protein lysates from untreated and UVC-irradiated (50 J/m^2^) MCF7 cells (Fig 2A, compare lanes 4 and 5).

### MDM2-ALT1 contributes to cell cycle arrest in a p53 and p21-dependent manner

Because over-expression of MDM2-ALT1 and MDMX-ALT2 leads to the accumulation of wild-type p53 protein in MCF7 cells, it raises the possibility that transcriptional targets of p53 may also be upregulated in response to the expression of these stress-inducible splice forms. We therefore examined the expression of *p21* (Cyclin-dependent kinase inhibitor 1), a transcriptional target of p53 [53], in MCF7 cells expressing LacZ, MDM2-ALT1 or MDMX-ALT2. Indeed, we observed a significant upregulation of *p21* at the transcriptional level in cells expressing MDM2-ALT1 compared to LacZ expression (Fig 2B). In the case of MDMX-ALT2 over-expressing cells, the rise in *p21* transcript levels was not statistically significant (Fig 2B). This pattern is consistent with the moderate rise in p53 levels observed upon MDMX-ALT2 over-expression (Fig 2A p53 panel). However, the rise in p21 protein levels was evident both in cells transfected with MDM2-ALT1 and in cells expressing MDMX-ALT2 compared to cells expressing LacZ (Fig 2C, compare lanes 2 and 3 with lane 1). The UVC irradiated cells were used as a positive control of p21 induction compared to the untreated cells (Fig 2C, lanes 4 and 5).

**Figure 3:**
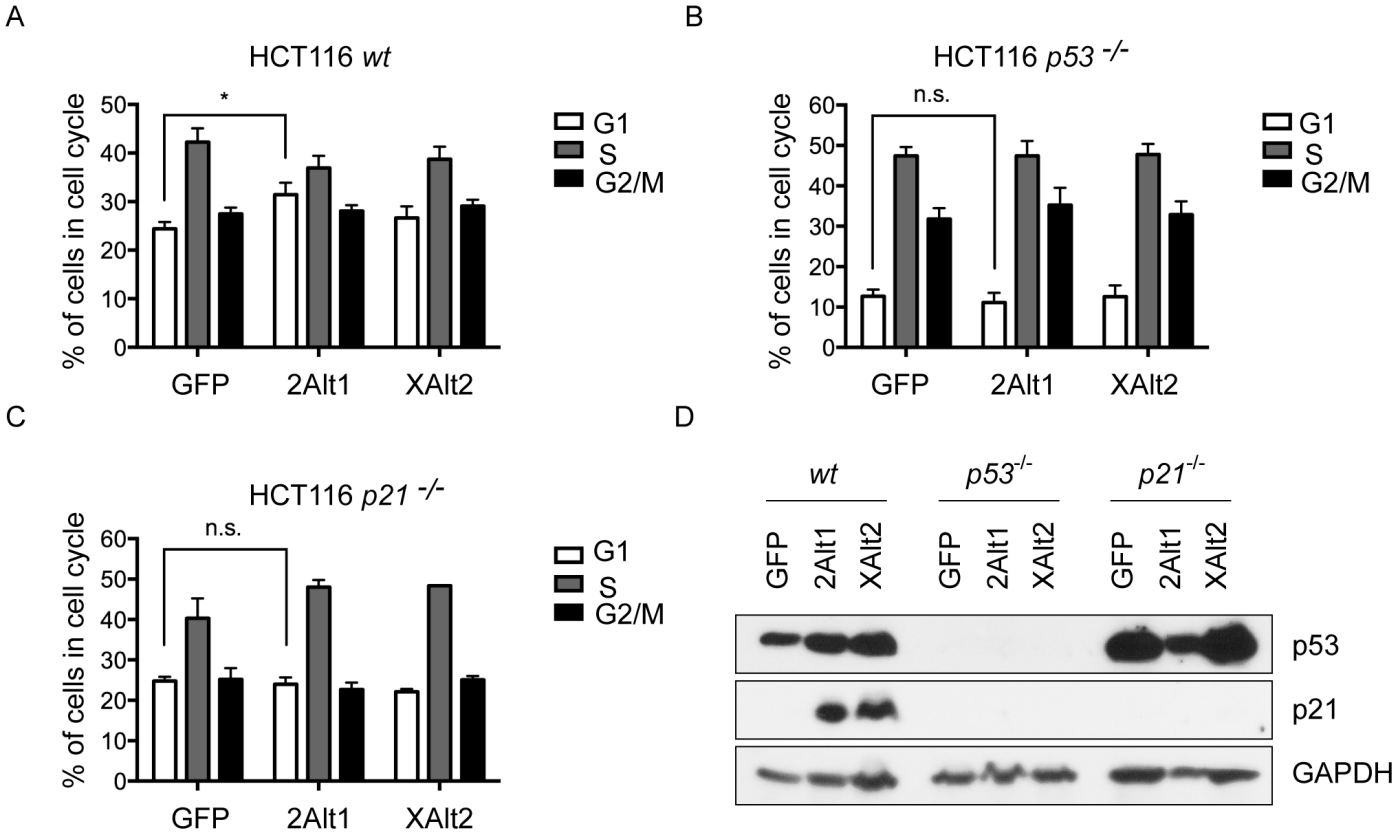
MDM2-ALT1 over-expression leads to G1 phase cell cycle arrest in a p53-dependent and p21-dependent manner. A. HCT116 wild-type (wt), B. HCT116 *p53* ^−/−^ and C. HCT116 *p21* ^−/−^ cells were transfected with myc-tagged GFP or MDM2-ALT1 (2Alt1) or MDMX-ALT2 (XAlt2), harvested 24 hours post-transfection and stained with propidium iodide solution and sorted for DNA content. The bar graphs represent the percentage of cells in the various phases of the cell cycle. Error bars represent the standard error mean from at least 3 independent experiments. HCT116 cells that are wildtype (A) show significantly higher percentage of cells in G1-phase upon MDM2-ALT1 expression (31.44% ±2.45 SEM) compared to GFP-expressing cells (24.40% ±1.40 SEM; n = 5, *p* = 0.0369). In case of MDMX-ALT2 over-expression in HCT116 wildtype cells, there is no significant change in percentage of cells in G1 phase compared to negative control GFP-expressing cells (*p* =  0.4389). HCT116 cells that are null for *p53* (B) or *p21* (C) do not show any differences in the percentage of cells in any of the cell cycle phases upon over-expression of GFP or MDM2-ALT1 or MDMX-ALT2. D. Representative immuno blot showing expression of p53, p21 and loading control GAPDH in HCT116 *wt*, *p53* ^−/−^ and *p21* ^−/−^ cells.

As p21 is a strong inhibitor of the transition from G1 to S phase during the cell cycle, we examined the effects of p21 upregulation on cell cycle progression in MCF7 cells expressing MDM2-ALT1 or MDMX-ALT2 in comparison with cells expressing LacZ. Propidium iodide staining of the transfected cells was used to identify the number of cells populating the various phases of the cell cycle. We observed that the percentage of cells in G1 phase was significantly higher in samples expressing MDM2-ALT1 compared to LacZ expressing samples in a manner concordant with the upregulation of p21 levels upon MDM2-ALT1 expression (Fig S3A). However, this difference was not significant in the cells expressing MDMX-ALT2 (Fig S3A). These results indicate that upon MDM2-ALT1 expression at least, the upregulation of p21 resulting from the stabilization of p53 can have functional consequences as evidenced by the G1-phase cell cycle arrest in cells expressing MDM2-ALT1. To determine whether the effect on cell cycle progression is p53-dependent, we utilized H1299 cells that are p53-null and show that the over-expression of neither MDM2-ALT1 nor MDMX-ALT2 results in G1 phase cell cycle arrest (Fig S3B). These experiments establish the p53-dependence of MDM2-ALT1 mediated G1 phase cell cycle arrest (compare the effects on MCF7 cells with wild-type p53 and H1299 cells, which are p53-negative). However, it is possible that these effects arise from genetic differences between the breast carcinoma (MCF7) and non small-cell lung carcinoma (H1299) cell lines. To rule out such a possibility, we over-expressed GFP or MDM2-ALT1 or MDMX-ALT2 in HCT116 cells that are either wild-type (Fig 3A) or null for p53 (Fig 3B). Indeed, even in these isotype matched cell-lines, we observed that the MDM2-ALT1 mediated G1 phase cell cycle arrest is dependent on the presence of p53 as only HCT116 cells wild-type for p53 (Fig 3A) and not those null for p53 (Fig 3B), showed significantly higher percentage of cells stalled in G1 phase compared to GFP expressing cells. Here also the expression of MDMX-ALT2 did not show significant changes in cell cycle progression compared to GFP (Fig 3).

To examine whether or not the G1 phase cell-cycle arrest observed in the HCT116 cells upon MDM2-ALT1 over-expression is p21 dependent, we compared HCT116 wild-type and p21-null cells under conditions of over-expression of GFP, MDM2-ALT1 or MDMX-ALT2. Indeed, we observed that the loss of p21 abolished the cell-cycle arrest phenotype induced by MDM2-ALT1 over expression (Fig 3C). The HCT116 p21-null cells transfected with GFP or MDM2-ALT1 or MDMX-ALT2 showed no statistically significant changes in the percentage of cells in any of the cell cycle phases. These results indicate the cell-cycle arrest induced by MDM2-ALT1 is p53 and p21 dependent. The expression of p53 and p21 in the various HCT116 cell-lines were confirmed by western blot (Fig 3D).

### MDM2-ALT1 and MDMX-ALT2 lead to activation of distinct p53 transcriptional targets

**Figure 4:**
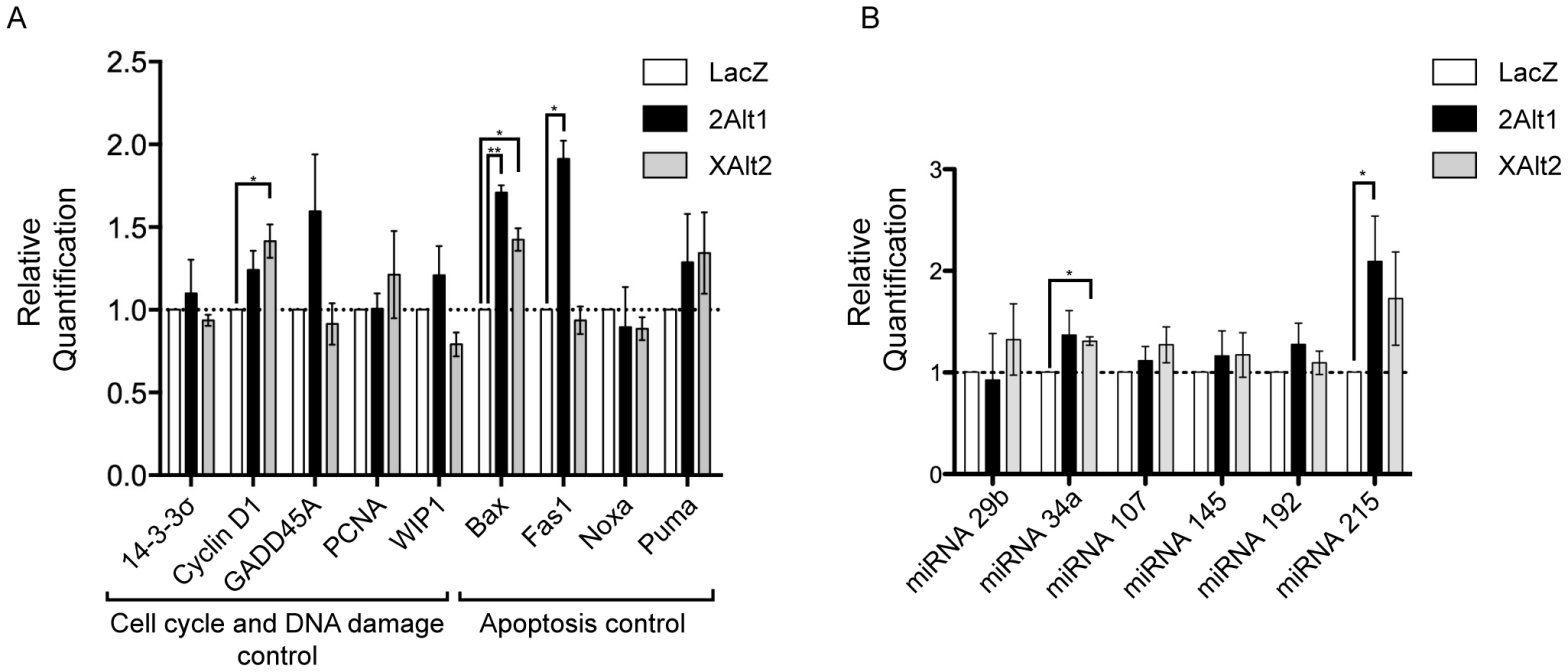
MDM2-ALT1 and MDMX-ALT2 lead to the activation of subtly different subsets of p53 transcriptional targets. **A.** MCF7 cells transfected with myc-tagged LacZ, or MDM2-ALT1 (Alt1) or MDMX-ALT2 (XAlt2) were examined for mRNA levels of a panel of 9 p53 transcriptional targets using quantitative real-time PCR and were normalized to LacZ-expressing cells. The bar graphs represent the relative quantification (2^−ΔΔCt^) values from at least 3 independent experiments and each consisting of 3 technical replicates. The error bars represent standard error means (SEM). **B.** qRT-PCR was used to compare the relative levels (2^−ΔΔCt^) of miRNA targets of p53 between cells over-expressing LacZ or MDM2-ALT1 or MDMX-ALT2. Here also a minimum of 3 independent trials was performed with 3 technical replicates each. The error bars represent standard error means (SEM). * represents *p*<0.05 and ** represents *p*<0.01 in all cases.

Since the over-expression of MDM2-ALT1 and MDMX-ALT2 lead to the upregulation of p53-target p21, we hypothesized that other transcriptional targets of p53 could also be upregulated under these circumstances. We assessed the transcript levels of candidate genes involved in the p53 tumor suppressor pathway and known to play roles in either cell-cycle control and/or DNA damage repair (*p21*, *GADD45A*, *WIP1*, *PCNA*, *Cyclin D1* and *14-3-3σ*) or apoptosis (*Bax*, *Fas1*, *PUMA*, *Noxa*) [54]–[66]. Of the 9 additional p53-target genes whose expression was assayed, only 3 (*Bax*, *Cyclin D1*, and *Fas1*) showed a significant increase in response to MDM2-ALT1 or MDMX-ALT2 over-expression. *Bax* transcript levels were significantly upregulated in response to the over-expression of MDM2-ALT1 as well as MDMX-ALT2 (Fig 4A). However, *Cyclin D1* levels showed significant increase only upon MDMX-ALT2 expression and *Fas1* transcripts were significantly higher only in cells over-expressing MDM2-ALT1. In all cases the transcript levels of the p53-target genes upon MDM2-ALT1 or MDMX-ALT2 over-expression were normalized to corresponding transcript levels in LacZ expressing cells and *GAPDH* mRNA was used as the endogenous control (Fig 4A).

Similarly, we also examined the expression of 6 miRNA targets of p53 including miRNAs 34a, 107, 145, 192, 215, and miR29b [67]–[75]. While most miRNA targets show moderate increase in expression after MDM2-ALT1 or MDMX-ALT2, only miRNA 215 or miRNA 34a levels were significantly increased in cultures expressing MDM2-ALT1 or MDMX-ALT2, respectively (Fig 4B). The transcript levels of the miRNA targets of p53 were normalized to *U6 snRNA* endogenous control and then normalized again to the LacZ transfected controls in order to obtain the relative expression of the miRNA targets of p53. These results indicate that while MDM2-ALT1 and MDMX-ALT2 lead to increased p53 protein levels, the transcriptional activity of p53 is restricted to specific targets of p53 raising the possibility that these two splice forms act through distinct molecular pathways to regulate the p53 response.

To determine the p53 transcriptional effects that are mediated as a result of disrupted p53-MDM2 interaction in a manner similar to MDM2-ALT1 and MDMX-ALT2 over-expression, we used Nutlin-3, a drug that specifically inhibits the binding of p53 with MDM2. We assessed the expression of our panel of p53 targets from DMSO or Nutlin-3 treated cultures. We observed significant upregulation of *Bax*, *Fas1* and *p21* upon Nutlin-3 treatment when compared to DMSO treated cultures (Fig S4A). Importantly, these three targets are also upregulated after MDM2-ALT1 overexpression suggesting that overexpression of MDM2-ALT1 may induce transcription of these targets via an overlapping mechanism with Nutlin-3, specifically the disruption the p53-MDM2 interaction. In contrast, *14-3-3σ* and *WIP1* were significantly upregulated under Nutlin-3 treatment but not upon over-expression of either MDM2-ALT1 or MDMX-ALT2 (Fig S4A). Surprisingly, none of the miRNA targets showed a significant change in expression upon Nutlin-3 treatment (Fig S4B) despite showing an increase in p53 (Fig S4C). Our results indicate that, though there is some overlap in transcriptional target activation with Nutlin-3 treatment and overexpression of MDM2-ALT1, there are likely p53-independent mechanisms for MDM2-ALT1 transcriptional activation as well.

## Discussion

### Stress-responsive *MDM2* and *MDMX* alternative splicing

MDM2 and MDMX possess distinct, non-overlapping roles in p53 regulation. Misregulation of alternative splicing in various types of cancers generates different isoforms of MDM2 and MDMX, some of which have been shown to affect protein-protein interaction and tumor suppressor activity of p53. In this study, we focused on two such cancer-associated splice forms MDM2-ALT1 and MDMX-ALT2 that are also coordinately generated in cells in response to genotoxic stress including UV and Cisplatinum [29], [43].

Both, MDM2-ALT1 and MDMX-ALT2 lack the p53-binding domain but they retain the RING finger domain [29], [43]. The RING domain of MDM2 is not only required for E3 ubiquitin ligase activity but also for its heterodimerization with MDMX and further fine-tuning of self and target protein ubiquitination and degradation [6], [14], [15], [76]–[78]. Consistent with previous observations, our current work demonstrated that MDM2-ALT1 interacts with endogenous MDM2 (Fig 1C), but we also observed a robust interaction of MDM2-ALT1 with endogenous MDMX (Fig 1D). Moreover, MDMX-ALT2 interacted with endogenous MDMX and also with MDM2 (Fig 1C, D). These results confirm that MDM2-ALT1 heterodimerizes with endogenous MDMX and MDMX-ALT2 forms a heterodimer with endogenous MDM2. Hence, we hypothesized that perturbed stoichiometry of endogenous MDM2/MDMX by overexpression of MDM2-ALT1 or MDMX-ALT2 would lead to stabilization of p53, MDM2, and MDMX. We observed that p53 and MDM2 were stabilized but the overall levels of MDMX remained unchanged suggesting that MDM2-ALT1 or MDMX-ALT2 does not seem to affect MDMX.

Despite a moderate increase in *p21* transcripts (a p53 target gene) after overexpression of MDMX-ALT2 (Fig 2B), the p21 protein levels were comparable to that of expression of MDM2-ALT1 (Fig 2C). This raises the possibility that MDMX-ALT2 could play a role in modulating p21 protein stability. Both full-length MDM2 [79], [80] and MDMX [81] have been shown to promote the degradation of p21 in a p53 and ubiquitination independent manner. As MDMX-ALT2 interacts with endogenous MDM2 and MDMX, it is possible that it impedes the MDM-mediated degradation of p21 thereby leading to increased stabilization of p21. Hence, it is possible that MDM2-ALT1 and MDMX-ALT2 can also lead altered stability of other protein targets of MDM2 and MDMX including p21.

### Effects of the expression of MDM2 splice variants on cell cycle

Because upregulation of p53 and generation of various isoforms of MDM2 and MDMX occur after exposure to genotoxic stress, we wanted to test the effects of the overexpression of predominant isoforms MDM2-ALT1 and MDMX-ALT2 on p53 levels. Our current data suggest that overexpression of MDM2-ALT1 or MDMX-ALT2 is sufficient to induce upregulation of p53 and MDM2 (Fig 1C and 2A-C). Next, we also determined the effect of increased p53 after overexpression of MDM2-ALT1 or MDMX-ALT2 on cell cycle by propidium iodide staining (Fig 3). Concordant with the increased levels of p21, MDM2-ALT1 over-expression led to G1-S phase cell cycle arrest in these cells (Fig 3, S3). Moreover, this effect was found to be p53 and p21-dependent as the over-expression of MDM2-ALT1 in HCT116 p21 or p53 null cells abolished the cell-cycle arrest phenotype observed in HCT116 wild type cells expressing MDM2-ALT1. Despite similar levels of p21 in cells overexpressing MDM2-ALT1 or MDMX-ALT2, the overexpression of MDMX-ALT2 did not induce cell-cycle arrest suggesting requirement of additional events for induction of cell-cycle arrest. Interestingly, the growth inhibitory effect of expression of MDM2-A, another common cancer-associated *MDM2* splice variant, is independent of p21 although this splice variant also activates the p53 pathway and causes the upregulation of p21 [27], [28]. Another cancer-associated splice variant MDM2-ALT3 has so far been shown to inhibit the degradation of p53 and so it is likely that MDM2-ALT3 over-expression can also affect the cell cycle [26]. Both, *MDM2-ALT2* and *MDM2-ALT3* are also generated in response to genotoxic stress, albeit to a lesser extent [29]. Hence, it is possible that other alternative splice isoforms of MDM2 and MDMX work together with the predominant isoforms MDM2-ALT1 and MDMX-ALT2 to modulate p53 activity, cell growth and function after genotoxic stress.

### Altered expression of p53 targets after expression of MDM2-ALT1 or MDMX-ALT2 splice variants

In response to genotoxic stress p53 is upregulated and posttranslationally modified which aids in recruitment of chromatin modifying/remodeling factors to activate p53 target gene transcription [82]–[86]. Moreover, the transcriptional output of p53 target genes determines the type and extent of the DNA-damage response induced. It should be noted that stress stimuli activate multiple tumor-suppressor and DNA-damage signaling networks that feed into the p53 pathway and would occlude a clear understanding of the roles of MDM2-ALT1 or MDMX-ALT2 in this scenario. We wanted to identify the overlap of p53 target mRNA and miRNA expression changes mediated by overexpression of MDM2-ALT1 or MDMX-ALT2 with those affected by disrupting MDM2-p53 interaction, because overexpression of these isoforms also alters MDM2-p53 interaction. Nutlin-3, on the other hand specifically inhibits the interaction of MDM2 and p53 thereby causing the stabilization of p53. An assessment of mRNA and miRNA targets of p53 by quantitative RT-PCR indicated that MDM2-ALT1 and MDMX-ALT2 modulate p53 transcriptional activity in distinct ways. We also observed that MDM2 protein levels were upregulated as a result of the over-expression of MDM2-ALT1 or MDMX-ALT2 possibly because *MDM2* is a transcriptional target of p53. Upregulation of *Bax* was observed after expression of both MDM2-ALT1 and MDMX-ALT2 suggesting that upregulation of p53 mediated by these splice forms is sufficient to induce expression of *Bax*, whereas productive transcription of other p53 target genes requires additional components of genotoxic stress induced pathways. Similarly, miRNA 34a is significantly upregulated upon over-expression of MDMX-ALT2 (Fig 4B), whereas upregulation of miRNA 215 is observed after MDM2-ALT1 expression (Fig 4B) suggesting differential activity of p53 under these conditions for expression of miRNA targets.

Apoptotic genes *Bax*, *Fas1* and cell-cycle control genes *GADD45A* and *p21* were upregulated under both conditions of MDM2-ALT1 or MDMX-ALT2 over-expression and Nutlin-3 treatment. However, the fold changes in the expression of the individual target genes observed upon Nutlin-3 treatment were much higher. One possible reason could be greater efficacy of drug delivery compared to transfection efficiency of cells. There were also differences between MDM splice variant over-expression and Nutlin-3 treatment. While Nutlin-3-induced p53 caused significant upregulation of *14-3-3σ*, *Puma* and *WIP1*, neither MDM2-ALT1 nor MDMX-ALT2 contributed to changes in the expression of these genes. On the other hand, neither Nutlin-3 treatment nor over-expression of MDM2-ALT1/MDMX-ALT2 led to a significant change in expression of *Noxa* and *PCNA* suggesting additional molecular requirements for activation of a subset of p53 target genes i.e. posttranslational modification of p53 and/or presence of co-activators. Interestingly, none of the miRNA targets of p53 showed changes in expression levels upon Nutlin-3 treatment. This is in contrast with previous studies examining Nutlin-3 treatment of cells where the expression of at least miRNAs 34, 192 and 215 were shown to be altered in response to Nutlin-3 [71]. However, it is possible that cell line differences could account for these discrepancies. Additionally p53 shares target genes with its family members p63 and p73 whose levels and activity are also regulated in a complex manner by MDM2 and MDMX and it is possible that MDM2-ALT1 and MDMX-ALT2 influence these interactions as well. However, these possibilities are yet to be extensively explored.

### MDM2 and MDMX splicing in cancer

**Figure 5:**
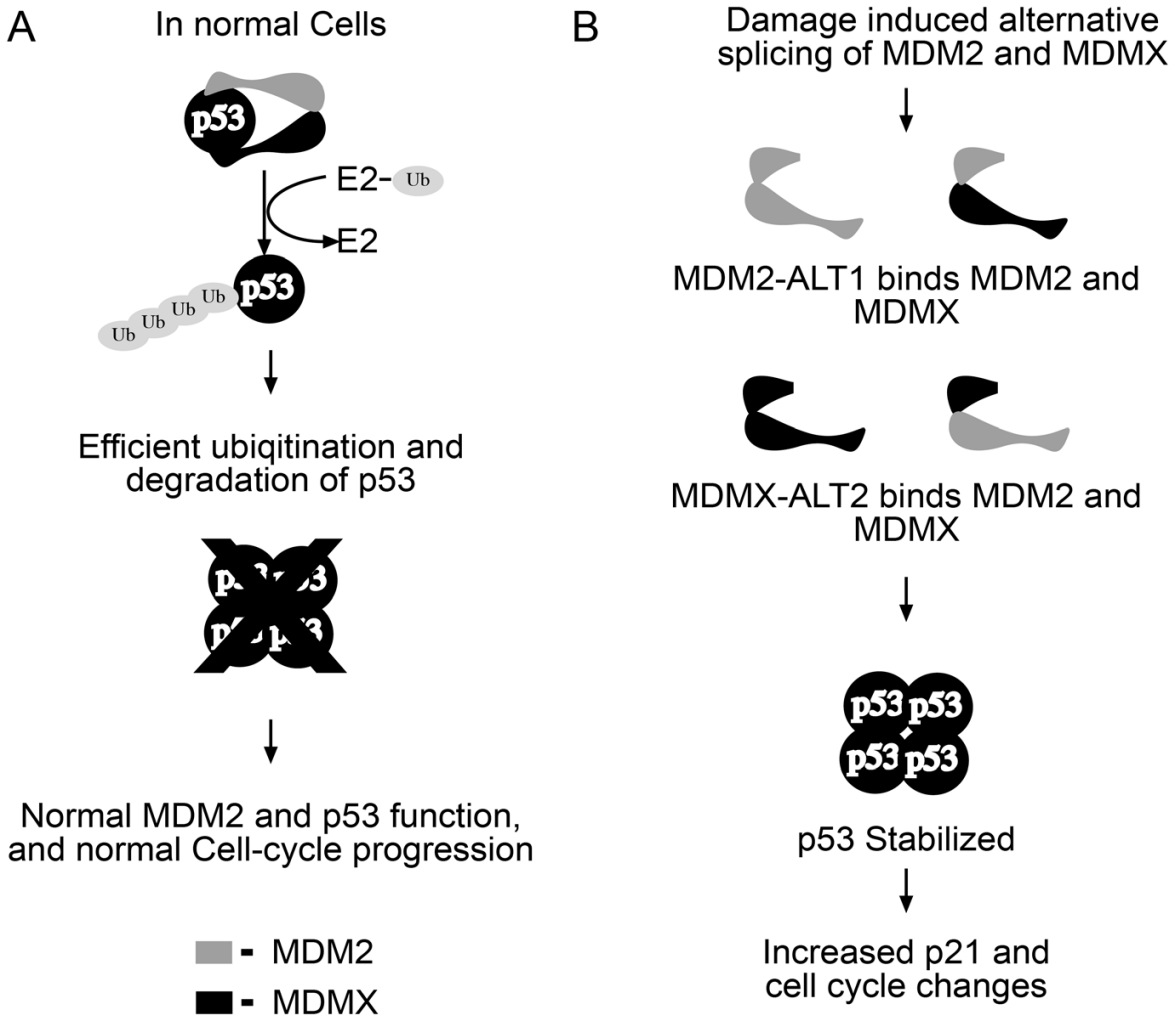
Proposed model: MDM2-ALT1 and MDMX-ALT2 antagonize their full-length counterparts and lead to p53 stabilization. A. Under normal conditions, MDM2 and MDMX function to maintain low levels of p53 (via ubiquitination and subsequent degradation) and curb its transcriptional activity by binding p53. This helps maintain homeostasis and normal cellular functions including cell cycle progression. B. Under genotoxic stress, alternative splice forms MDM2-ALT1 and MDMX-ALT2 interact with the full-length MDM proteins and interfere in their p53-regulatory functions. This leads to the stabilization and upregulation of p53 levels and also the activation of p53 transcriptional targets leading to changes in cell cycle progression.

Overall, we present our findings in the following model: under normal conditions when the alternative splice forms such as MDM2-ALT1 and MDMX-ALT2 are absent, their full-length MDM2 and MDMX functional normally to inhibit p53 activity (Fig 5A). However, under DNA damaging conditions and in cancers, the splice variants like MDM2-ALT1 and MDMX-ALT2, or other splice variants as discussed above, interact with and cause altered activity of endogenous MDM2 and MDMX leading to the stabilization of p53 and also act to fine-tune p53 transcriptional activity (Fig 5B).

Recently, overexpression of MDM2-ALT1 has been correlated with accumulation of mutant p53 in colorectal tumors [32], however previous work from our lab has demonstrated accumulation of wild-type p53 in RMS tumors expressing MDM2-ALT1 [43]. A critical finding of our work is that in addition to interacting with their endogenous counterparts, MDM2-ALT1 and MDMX-ALT2 heterodimerize with endogenous MDMX and MDM2, respectively, in effect debilitating both the formidable negative regulators of p53. Therefore, expression of either MDM2-ALT1 or MDMX-ALT2 would lead to increased p53 levels to allow tumorigenesis (in the case of p53 with dominant mutations) or tumor suppression (in the case of wild-type p53). The discovery of a diverse array of p53 transcriptional activity upon over-expression of MDM2-ALT1 or MDMX-ALT2 suggests further fine-tuning of p53 in conditions where these isoforms are expressed. Interestingly, multiple RMS tumors co-express *MDM2-ALT1* and *MDMX-ALT2* and also show accumulation of wild-type p53 suggesting that tumor suppressor activity is subsequently rendered ineffective in these tumors [43]. Besides interacting with p53, both MDM2 and MDMX regulate multiple aspects of gene expression by their interaction with other proteins [87]–[104]. It will be of interest to determine if MDM2-ALT1 and MDMX-ALT2 play p53-independent roles in activation of gene regulatory programs that promote tumor growth or survival in RMS and other tumor types that express these spliced isoforms. Our work has previously demonstrated the expression of MDM2-ALT1 and MDMX-ALT2 upon exposure to genotoxic stress [29], [43]. Further research examining the signaling events and factors that regulate normal splicing of *MDM2* and also the misregulated splicing observed in cancers will be critical for identifying therapeutic strategies for treatment of tumors harboring aberrantly spliced isoforms of MDM2 and MDMX.

## Materials and Methods

### Cell culture, transfections and over-expression constructs

MCF7 (breast carcinoma, ATCC HTB-22) and H1299 (non-small cell lung carcinoma, ATCC CRL-5803) cells were cultured under standard conditions at 37°C in DMEM with high glucose supplemented with 10% Fetal Bovine Serum (FBS, Hyclone, Logan UT), L-Glutamine (Cellgro, 25-005 CI) and Penicillin-Streptomycin (Cellgro, 30-001 CI). HCT116 (colorectal) cells that are wildtype or p53 null or p21 null [105] were cultured in McCoy's media supplemented with 10% FBS, L-Glutamine and Penicillin-Streptomycin. Transfection of the over-expression constructs myc-tagged LacZ, MDM2-ALT1 or MDMX-ALT2 [43] was performed using Fugene 6 (Promega, Madison, WI) according to manufacturer's instructions. Cells were typically harvested 24 hours post-transfection for protein or RNA. The over-expression of the myc-tagged proteins was confirmed by immunoblotting in every experiment.

### Co-immunoprecipitation and Immunoblotting

For the immunoprecipitation (IP) of myc-tagged proteins LacZ or MDM2-ALT1 or MDMX-ALT2 and also for the reciprocal IP of endogenous MDM2, MCF7 cells were transfected with the corresponding over-expression plasmids and 24 hours post-transfection, the cells were harvested in NP40 protein lysis buffer. Equal amounts of total protein (1000–2500 µg) were used for the immunoprecipitation of the myc-tagged proteins or endogenous MDM2. Briefly, 500 µl of precleared protein lysate (30 minutes on ice with 5 µg normal mouse IgG sc-2343AC, Santa Cruz Biotechnology) was incubated overnight at 4°C with anti-myc tag antibody (conjugated to agarose beads, sc-40 9E10AC, Santa Cruz Biotechnology) or the anti-MDM2 SMP14 antibody (conjugated to agarose beads, sc-965AC, Santa Cruz Biotechnology) at concentration of 1 µg primary antibody per 100 µg protein. Following immunoprecipitation, the beads were collected, washed, boiled in 2X SDS loading buffer and equal volumes of the eluate containing the immunoprecipitated proteins were separated on 10% SDS-PAGE gels. For the IP input and all other immunoblotting analyses, 30 µg of the protein lysate was separated on 10% SDS-PAGE gels. Antibodies used for immunoblotting were as follows: anti-MDM2 (2A10 mAb: epitope amino acids 294 to 339), kind gift of Dr. Lindsey Mayo, Indiana University and SMP14 sc-965 Santa Cruz Biotechnology: epitope amino acids 154-167), anti-MDMX (1 µg/ml, A300-287A, Bethyl Laboratories, Montgomery, TX: epitope amino acids 125 to 175 of MDMX), anti c-Myc (0.2 µg/ml, clone 9E10, sc-40 or clone A-14, sc-789, Santa Cruz Biotechnology), anti-p53 (0.2 µg/ml, DO-1 clone, sc-126, Santa Cruz Biotechnology), anti-p21 (0.2 µg/ml, F-5, sc-6246, Santa Cruz Biotechnology), β-Actin (AC-15, A5441, Sigma Aldrich, St. Louis, MO) and GAPDH (14C10, catalog no. 2118, Cell Signaling).

### Cell Cycle analyses and Flow Cytometry

MCF7 and H1299 cells were transfected with plasmids expressing myc tagged LacZ or GFP or MDM2-ALT1 or MDMX-ALT2 using Fugene 6. 24 hours post-transfection, the cells were collected and fixed in 70% ethanol. The cells were then stained with propidium iodide staining solution (100 U/ml RNAse A and 50 µg/ml propidium iodide in PBS) for 30 minutes. Following this the cells were sorted for their DNA content using BD LSR II system (BD Biosciences, San Jose, CA) and data analysis was performed using FloJo software (Treestar Inc., Ashland OR). The cell cycle data were then analyzed using one-way ANOVA with the Newman-Keul's Multiple Comparison post-test on the GraphPad Prism software (version 5.0). The HCT116 cells (wildtype, p53 null and p21 null) were nucleofected with the corresponding expression constructs using the Amaxa Cell Line Nucleofector Kit V (Lonza, Cologne, Germany) according to manufacturer's instructions. The cells were then stained with propidium iodide staining solution (100 U/ml RNAse A, 5 mM EDTA pH 8.0 and 50 µg/ml propidium iodide in PBS) for 30 minutes. Following this the cells were sorted for their DNA content using BD LSR II system (BD Biosciences, San Jose, CA) and data analysis was performed using FloJo software (Treestar Inc., Ashland OR). The cell cycle data were then analyzed using unpaired, two-tailed Student's T-Test on the GraphPad Prism software (version 6.0).

### Reverse Transcription and quantitative Real-Time Polymerase Chain Reaction (qRT-PCR)

The MCF7 cells were transfected with myc-tagged LacZ or MDM2-ALT1 or MDMX-ALT2 expression constructs for 24 hours and were then harvested for RNA and protein. For the Nutlin-3 experiments, MCF7 cells were transfected with LacZ expressing plasmids and 24 hours post-transfection, were treated with 10µM Nutlin-3 (N6287-1MG, Sigma Aldrich) or equal volume of DMSO for 12 hours. They were then harvested for RNA and protein. RNA isolation was performed using the RNeasy Mini Protocol (Qiagen, Valencia, CA). Typically, 1 µg of RNA with random hexamers was used to synthesize cDNA in reverse transcription reactions that were carried out using the Transcriptor RT enzyme (Catalog no. 03531287001, Roche Diagnostics, Indianapolis, IN) according to manufacturer's instructions. For detection of miRNA targets, the reverse transcription reaction was performed using 1 µg of RNA with gene-specific reverse primers for either the p53-target miRNAs or for the *U6 snRNA* endogenous control. These primers are listed below. Real-time PCR reactions were carried out using the SYBR Green PCR master mix (Applied Biosystems part no. 4309155). For the real-time quantification of *p21* transcripts, the PCR reactions were performed on an ABI Prism 7500 Sequence Detection system (Applied Biosystems, Foster City, CA) at 50°C for 2 minutes, 95°C for 10 minutes with 40 cycles of 95°C for 1 minute and 60°C for 1 minute each. All other real-time PCR assays were performed on an ABI 7900HT Fast Real Time PCR system under reaction conditions of 50°C for 2 minutes, 95°C for 10 minutes with 40 cycles of 95°C for 15 seconds and 60°C for 1 minute each for the mRNA targets and for 50 cycles for the miRNA targets. *GAPDH* (for mRNA targets) or *U6 snRNA* (for miRNA targets) levels in each sample were used as the endogenous control detector in all cases. The primers used to amplify the various p53-target transcripts (mRNA and miRNA targets) are listed below. For the miRNA targets, the gene-specific reverse primers were used only for reverse transcription reaction and the PCR amplification was carried out using the specific forward primer and a universal reverse primer (listed below). *U6 snRNA* was amplified with the specific forward primer and the same reverse primer used for reverse transcription. All PCR reactions were carried out with 3 technical replicates and the amplification of single PCR products in each reaction was confirmed using dissociation curve analyses and also by separating the products of the real-time PCR in 2.5% agarose gels. In the case of *p21*, the ratio of its transcript levels to *GAPDH* was plotted using GraphPad prism (ver 5.0b) with error bars representing the values from 4 independent experiments. The levels of all other transcriptional targets of p53 in MDM2-ALT1 or MDMX-ALT2 expressing cells relative to the corresponding transcript levels in LacZ transfected cells was determined as the relative quantification (RQ) or the 2^−ΔΔCt^ values using the RQ manager (1.2.1) software. RQ values from a minimum of 3 independent experiments were plotted using GraphPad Prism (ver 6.0) and error bars represent the standard error mean (SEM) unless specified. Statistical analyses to compare the level of *p21* transcripts between LacZ over-expressing samples and the MDM2-ALT1 or MDMX-ALT2 expressing samples were performed using one-way ANOVA with Bonferroni's multiple comparison tests on the GraphPad Prism version 5.0b software. In all other cases, to determine statistical significance in the difference in transcript levels between the LacZ and MDM2-ALT1 or MDMX-ALT2 transfected groups, unpaired two-tailed students' T-tests were performed using GraphPad Prism ver 6.0.

### mRNA targets

*14-3-3σ:* Forward: 5′ GGCCATGGACATCAGCAAGAA 3′, Reverse: 5′ CGAAAGTGGTCTTGGCCAGAG 3′

*Bax:* Forward: 5′ CCCCGAGAGGTCTTTTTCCG 3′, Reverse: 5′ GGCGTCCCAAAGTAGGAGA 3′

*Cyclin D1:* Forward: 5′ CCCGCACGATTTCATTGAAC 3′, Reverse: 5′ AGGGCGGATTGGAAATGAAC 3′

*Fas1:* Forward: 5′ GGGGTGGCTTTGTCTTCTTCTTTTG 3′, Reverse: 5′ ACCTTGGTTTTCCTTTCTGTGCTTTCT 3′

*GADD45A:* Forward: 5′ GCTGGTGACGAATCCACATTC 3′, Reverse: 5′ CAGATGCCATCACCGTTCAGG 3′

*Noxa:* Forward: 5′ GCTGGAAGTCGAGTGTGCTA 3′, Reverse: 5′ CCTGAGCAGAAGAGTTTGGA 3′

*PCNA:* Forward: 5′ AGGCACTCAAGGACCTCATCA 3′, Reverse: 5′ GAGTCCATGCTCTGCAGGTTT 3′

*Puma:* Forward: 5′ CCCTGGAGGGTCCTGTACAA 3′, Reverse: 5′ CTCTGTGGCCCCTGGGTAA 3′

*WIP1:* Forward: 5′ GTTCGTAGCAATGCCTTCTCA 3′, Reverse: 5′ CAATTTCTTGGGCTTTCATTTG 3′

*p21:* Forward: 5′ CCTGTCACTGTCTTGTACCCT 3′, Reverse: 5′ GCGTTTGGAGTGGTAGAAATCT 3′

*GAPDH:* Forward: 5′ GATGCTGGCGCTGAGTACG 3′, Reverse: 5′ GCTAAGCAGTTGGTGGTGC 3′

### miRNA targets

Universal reverse primer: 5′ TGGTGTCGTGGAGTCG 3′

*miRNA 29b:* Forward: 5′ ACACTCCAGCTGGGTAGCACCATTTGAAATC 3′, Reverse: 5′ CTCAACTGGTGTCGTGGAGTCGGCAATTCAGTTGAGAACACTGA 3′

*miRNA 34a:* Forward: 5′ ACACTCCAGCTGGGTGGCAGTGTCTTAGCTG 3′, Reverse: 5′ CTCAACTGGTGTCGTGGAGTCGGCAATTCAGTTGAGACAACCAG 3′

*miRNA 107:* Forward: 5′ ACACTCCAGCTGGGAGCAGCATTGTACAGG 3′, Reverse: 5′ CTCAACTGGTGTCGTGGAGTCGGCAATTCAGTTGAGTGATAGCC 3′

*miRNA 145:* Forward: 5′ ACACTCCAGCTGGGGTCCAGTTTTCCCAGG 3′, Reverse: 5′ CTCAACTGGTGTCGTGGAGTCGGCAATTCAGTTGAGAGGGATTC 3′

*miRNA 192:* Forward: 5′ ACACTCCAGCTGGGCTGACCTATGAATTCAC 3′, Reverse: 5′ CTCAACTGGTGTCGTGGAGTCGGCAATTCAGTTGAGGGCTGTC 3′

*miRNA 215:* Forward: 5′ ACACTCCAGCTGGGATGACCTATGAATTGAC 3′, Reverse: 5′ CTCAACTGGTGTCGTGGAGTCGGCAATTCAGTTGAGGTCTGTC 3′

*U6 snRNA:* Forward: 5′ CTCGCTTCGGCAGCACA 3′, Reverse: 5′ AACGCTTCACGAATTTGCGT 3′

## Supporting Information

Figure S1 Myc-tagged MDM2-ALT1 and MDMX-ALT2 co-immunoprecipitate with endogenous full-length MDM2 and MDMX. Myc-tagged LacZ, GFP, MDM2-ALT1 or MDMX-ALT2 were immunoprecipitated using anti-myc tag antibody. The immunoprecipitated proteins were separated using SDS-PAGE and probed for **A**. endogenous MDM2 using SMP14 anti-MDM2 antibody. The lower panel confirms the over-expression of myc tagged proteins in the total cell lysate and also the immunoprecipitation of the myc-tagged proteins via anti-myc tag western blot. **B**. Myc tagged MDM2-ALT1 and MDMX-ALT2 co-immunoprecipitate with full-length MDMX (immuno blot using MDMX A300-287A antibody) but not myc-GFP or LacZ. The dashed boxes indicate the regions cropped in Figure 1C and 1D. Confirmation of immunoprecipitation of myc-tagged GFP, LacZ, MDM2-ALT1 and MDMX-ALT2 proteins via anti-myc tag immuno blot (lower panel).
(TIF)

Figure S2 Full-length MDM2 co-immunoprecipitates with myc-MDM2-ALT1 and myc-MDMX-ALT2. Reciprocal immunoprecipitation of full-length MDM2 using anti-MDM2 SMP14 antibody shows the specific interaction of MDM2 with myc-tagged MDM2-ALT1 and MDMX-ALT2 (immuno blot for anti-myc tag, upper panel). Representative blot from 3 independent experiments is depicted. The immunoprecipitation of MDM2 from cells transfected with myc-LacZ, MDM2-ALT1 and MDMX-ALT2 was confirmed by probing for full-length MDM2 using the anti MDM2 2A10 antibody (middle panel). Remnant signal from the c-Myc antibody caused the appearance of Myc-LacZ at higher exposures. GAPDH was used as loading control for the IP input samples (lowest panel).
(TIF)

Figure S3 MDM2-ALT1 over-expression leads to G1 phase cell cycle arrest in a p53-dependent manner. **A.** MCF7, **B**. H1299, cells were transfected with myc-tagged LacZ or MDM2-ALT1 (2Alt1) or MDMX-ALT2 (XAlt2), harvested 24 hours post-transfection and stained with propidium iodide solution and sorted for DNA content. The bar graphs represent the percentage of cells in the various phases of the cell cycle. Error bars represent the standard deviation from at least 3 independent experiments. * represents statistically significant results (*p*<0.05).
(TIF)

Figure S4 Expression of p53 target genes upon Nutlin-3 treatment shows similarities to MDM-ALT over-expression. MCF7 cells transfected with LacZ were treated with 10 µM Nutlin-3 or DMSO for 12 hours and then examined for the expression of **A**. p53 transcriptional target mRNAs and **B**. miRNA targets of p53 via quantitative real time PCR. The error bars represent SEM from at least 3 independent trials. * indicates statistically significant *p*<0.05 and ** indicates *p*<0.01. **C**. Representative immuno blot showing confirmation of p53 and p21 upregulation upon Nutlin-3 treatment compared to DMSO treated MCF7 cells. GAPDH was used as loading control.
(TIF)
