# Supplementary figures and images for: Stress-Induced Alternative Splice Forms of MDM2 and MDMX Modulate the p53-Pathway in Distinct Ways
Source: PLoS One. 2014 Aug 8;9(8):e104444. doi: 10.1371/journal.pone.0104444 (PMC4126728; doi:10.1371/journal.pone.0104444)

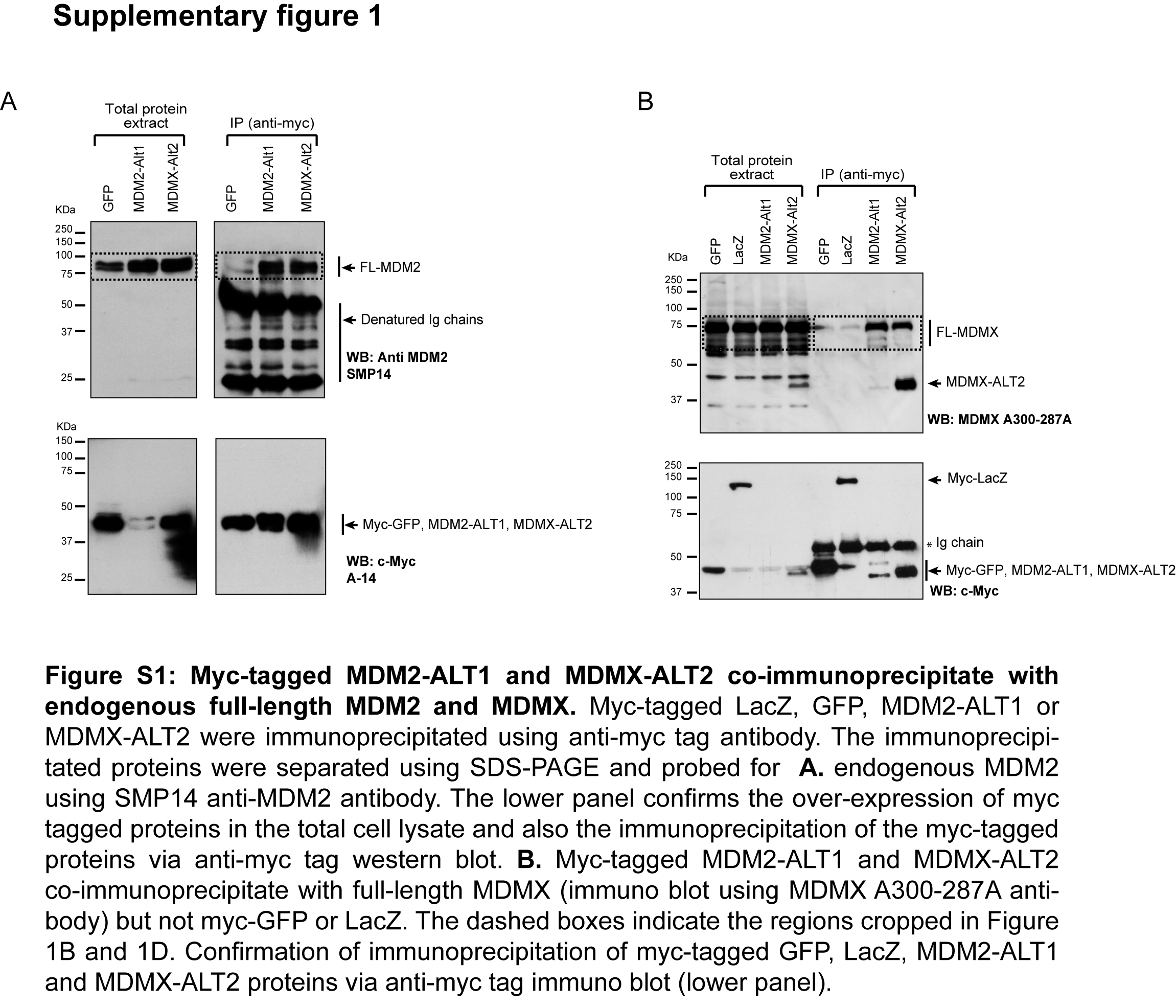

Supplement: Figure S1 — Myc-tagged MDM2-ALT1 and MDMX-ALT2 co-immunoprecipitate with endogenous full-length MDM2 and MDMX. Myc-tagged LacZ, GFP, MDM2-ALT1 or MDMX-ALT2 were immunoprecipitated using anti-myc tag antibody. The immunoprecipitated proteins were separated using SDS-PAGE and probed for A. endogenous MDM2 using SMP14 anti-MDM2 antibody. The lower panel confirms the over-expression of myc tagged proteins in the total cell lysate and also the immunoprecipitation of the myc-tagged proteins via anti-myc tag western blot. B. Myc tagged MDM2-ALT1 and MDMX-ALT2 co-immunoprecipitate with full-length MDMX (immuno blot using MDMX A300-287A antibody) but not myc-GFP or LacZ. The dashed boxes indicate the regions cropped in Figure 1C and 1D. Confirmation of immunoprecipitation of myc-tagged GFP, LacZ, MDM2-ALT1 and MDMX-ALT2 proteins via anti-myc tag immuno blot (lower panel). (TIF) [file pone.0104444.s001.tif]

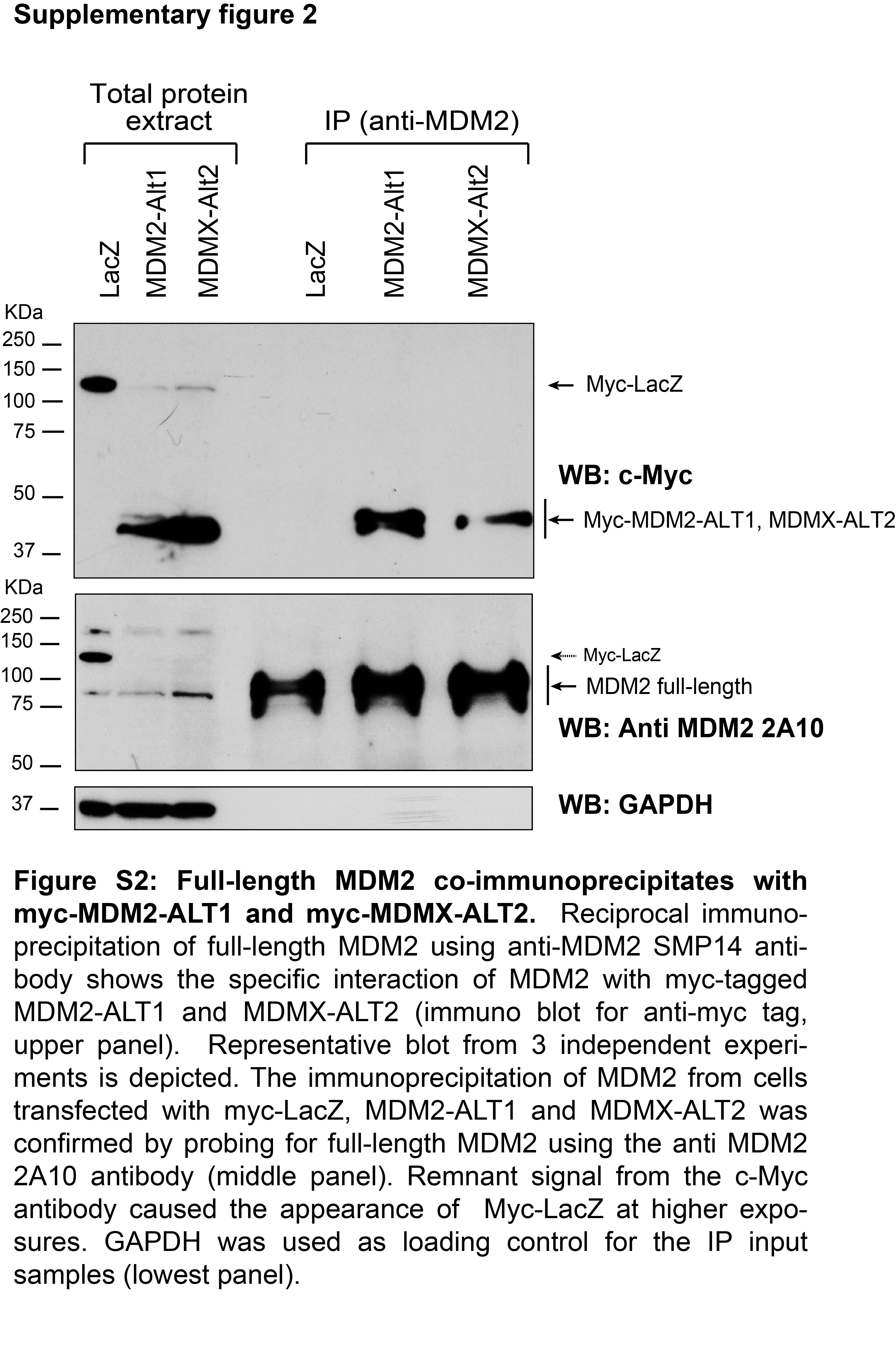

Supplement: Figure S2 — Full-length MDM2 co-immunoprecipitates with myc-MDM2-ALT1 and myc-MDMX-ALT2. Reciprocal immunoprecipitation of full-length MDM2 using anti-MDM2 SMP14 antibody shows the specific interaction of MDM2 with myc-tagged MDM2-ALT1 and MDMX-ALT2 (immuno blot for anti-myc tag, upper panel). Representative blot from 3 independent experiments is depicted. The immunoprecipitation of MDM2 from cells transfected with myc-LacZ, MDM2-ALT1 and MDMX-ALT2 was confirmed by probing for full-length MDM2 using the anti MDM2 2A10 antibody (middle panel). Remnant signal from the c-Myc antibody caused the appearance of Myc-LacZ at higher exposures. GAPDH was used as loading control for the IP input samples (lowest panel). (TIF) [file pone.0104444.s002.tif]

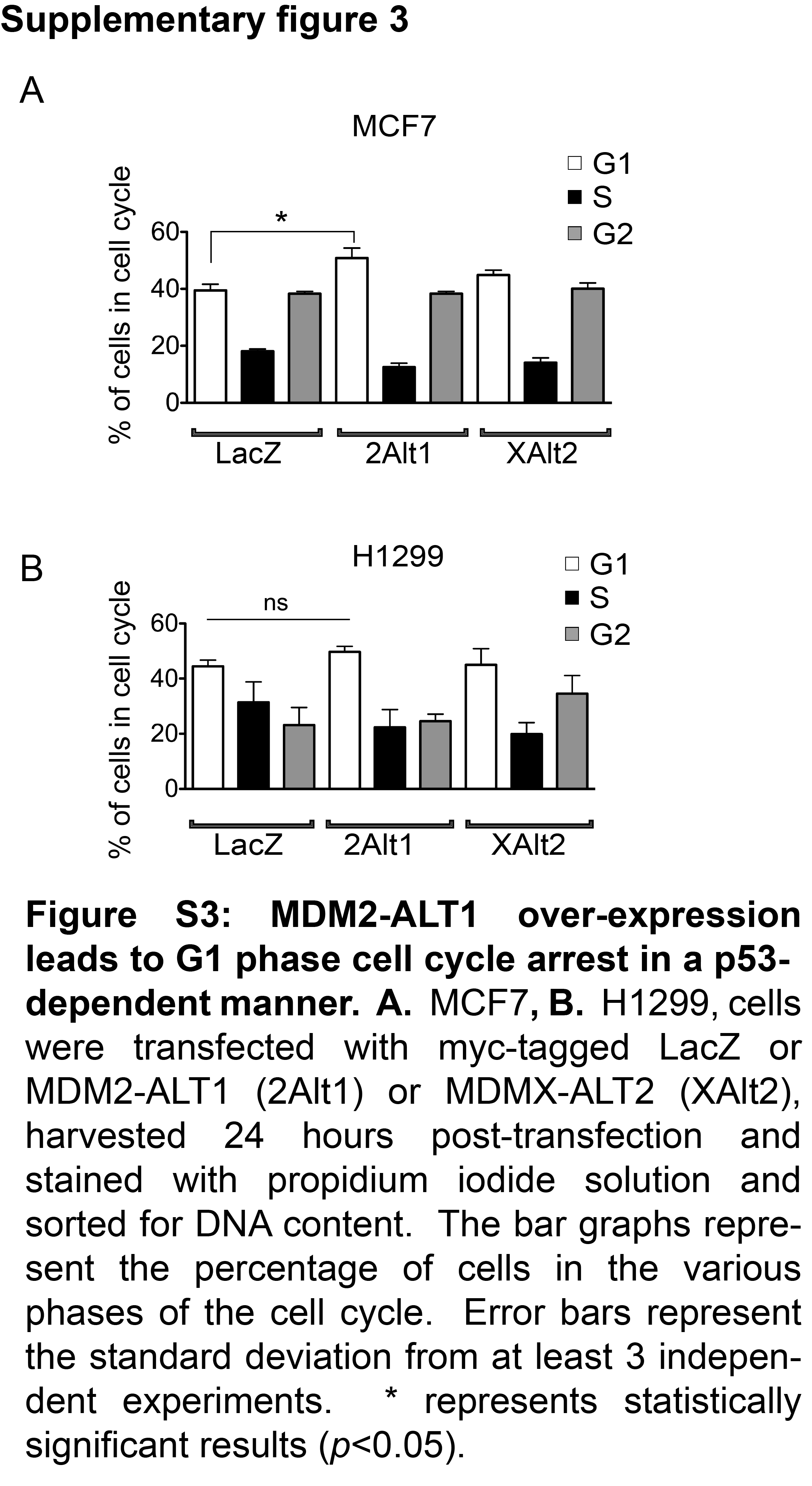

Supplement: Figure S3 — MDM2-ALT1 over-expression leads to G1 phase cell cycle arrest in a p53-dependent manner. A. MCF7, B. H1299, cells were transfected with myc-tagged LacZ or MDM2-ALT1 (2Alt1) or MDMX-ALT2 (XAlt2), harvested 24 hours post-transfection and stained with propidium iodide solution and sorted for DNA content. The bar graphs represent the percentage of cells in the various phases of the cell cycle. Error bars represent the standard deviation from at least 3 independent experiments. * represents statistically significant results (p<0.05). (TIF) [file pone.0104444.s003.tif]

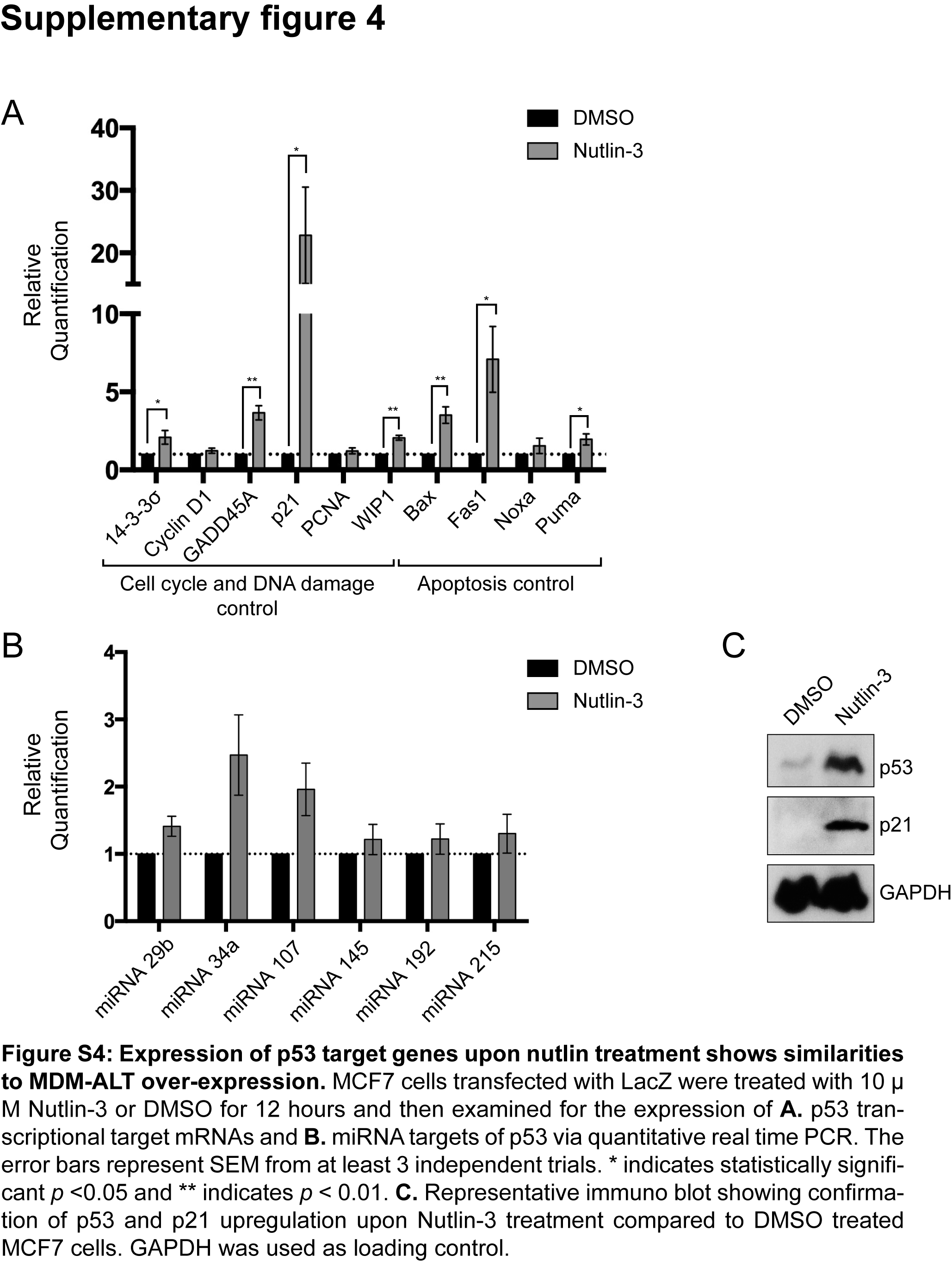

Supplement: Figure S4 — Expression of p53 target genes upon Nutlin-3 treatment shows similarities to MDM-ALT over-expression. MCF7 cells transfected with LacZ were treated with 10 µM Nutlin-3 or DMSO for 12 hours and then examined for the expression of A. p53 transcriptional target mRNAs and B. miRNA targets of p53 via quantitative real time PCR. The error bars represent SEM from at least 3 independent trials. * indicates statistically significant p<0.05 and ** indicates p<0.01. C. Representative immuno blot showing confirmation of p53 and p21 upregulation upon Nutlin-3 treatment compared to DMSO treated MCF7 cells. GAPDH was used as loading control. (TIF) [file pone.0104444.s004.tif]
